# Therapeutic Potential of Platelet-Derived Growth Factor B-Modified Nanoparticles in Enhancing Pericyte Activation for Acute Ischemic Stroke

**DOI:** 10.1523/ENEURO.0404-25.2026

**Published:** 2026-08-19

**Authors:** Satoshi Karashima, Koichi Arimura, Ryusuke Hatae, Soh Takagishi, Sayoko Narahara, Takahito Kawano, Shunya Tanaka, Ryota Kurogi, Akira Nakamizo, Masaharu Murata, Koji Yoshimoto

**Affiliations:** ^1^Department of Neurosurgery, Graduate School of Medical Sciences, Kyushu University, Fukuoka 812-8582, Japan; ^2^Center for Advanced Medical Innovation, Kyushu University, Fukuoka 812-8582, Japan; ^3^Department of Biomedical Engineering, Graduate School of Medical Science, Kyushu University, Fukuoka 812-8582, Japan

**Keywords:** ischemic stroke, nanoparticles, pericytes, platelet-derived growth factor subunit B (PDGF-B)

## Abstract

Platelet-derived growth factor subunit B (PDGF-B) signaling plays a critical role in pericyte activation and neurovascular repair following ischemic injury. Although enhancing PDGF-B signaling in pericytes represents a promising therapeutic strategy for ischemic stroke, practical approaches have not yet been developed. In this study, we evaluated the therapeutic efficacy and underlying mechanisms of PDGF-B-modified heat shock protein nanoparticles (PDGF-B HSPNPs) in a transient middle cerebral artery occlusion mouse model of either sex. Mice were treated with single intravenous injection of PDGF-B HSPNPs immediately after reperfusion, and infarct volume and motor function were assessed. To investigate cellular effects, flow cytometric analysis of brain-derived single–cell suspensions was performed 3 d after treatment to quantify platelet-derived growth factor receptor β-positive pericytes. Our results demonstrate that PDGF-B HSPNPs administration significantly reduced infarct volume and improved motor function, accompanied by enhanced activation of pericytes in the peri-infarct region. These findings suggest that PDGF-B HSPNPs represent a promising therapeutic approach for the treatment of ischemic stroke.

## Graphical abstract

**Figure:**
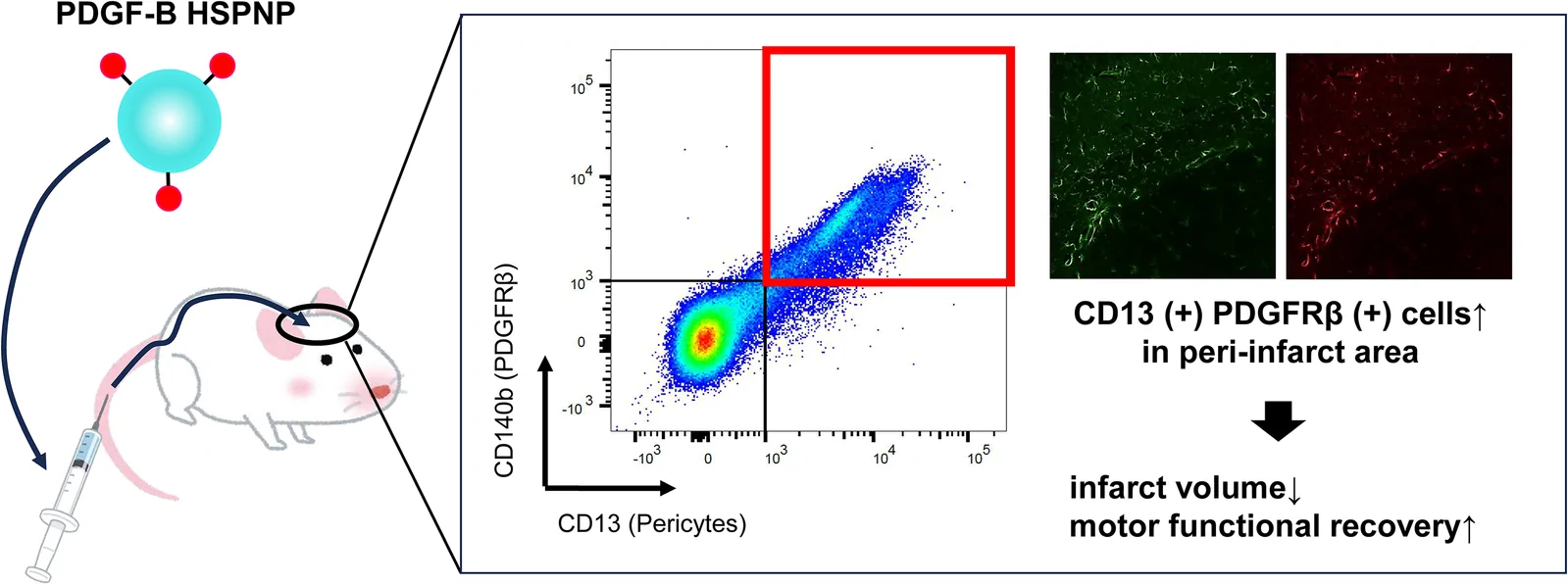
Visual Abstract

## Significance Statement

In this study, we demonstrated that immediate postreperfusion administration of platelet-derived growth factor subunit B-modified heat shock protein nanoparticles (PDGF-B HSPNPs) significantly improved the outcomes including the infarct volume and motor function recovery in a transient MCA occlusion mouse model. Importantly, our data revealed a possible mechanism by which PDGF-B HSPNPs enhance early activation of pericytes and endothelial cells—critical components of the neurovascular unit—leading to reduced infarct size and improved motor function. These findings suggest that nanoparticle-based targeting of pericyte pathways may represents a promising therapeutic strategy for acute ischemic stroke.

## Introduction

Ischemic stroke remains a leading cause of morbidity and mortality worldwide, often resulting in long-term disability due to irreversible neural injury (GBD, 2024). Although reperfusion therapies such as intravenous thrombolysis and mechanical thrombectomy have improved clinical outcomes, their application is limited by strict eligibility criteria (Goyal et al., 2016). Consequently, many patients still lack effective treatment options, highlighting the urgent need for novel neuroprotective strategies.

Pericytes, mural cells that envelop endothelial cells in capillaries and microvessels, are essential for maintaining blood–brain barrier (BBB) integrity, regulating cerebral blood flow, and facilitating neurovascular remodeling (Daneman et al., 2010; Armulik et al., 2011; Sweeney et al., 2016). Platelet-derived growth factor subunit B (PDGF-B) and its receptor PDGFRβ play critical roles in pericyte activation and recruitment following vascular injury (Lindahl et al., 1997; Arimura et al., 2012; Nakamura et al., 2016). Recent studies suggest that modulating PDGF-B/PDGFRβ signaling may provide neuroprotection in ischemic stroke by enhancing pericyte-mediated repair mechanisms (Shen et al., 2012; Makihara et al., 2015).

Takagishi et al. previously developed PDGF-B-modified heat shock protein nanoparticles (PDGF-B HSPNPs) and demonstrated that intravenous administration 24 h after transient middle cerebral artery occlusion (tMCAO) in mice significantly reduced infarct volume and motor deficits. These neuroprotective effects were associated with Akt (Ser473) phosphorylation, neurotrophin-3 (NT-3) upregulation, reduced neuronal apoptosis, and enhanced astrogliosis (Takagishi et al., 2021).

However, the cellular mechanisms underlying the efficacy of PDGF-B HSPNPs when administered immediately after reperfusion remain unclear. Conventional histological techniques may not adequately capture dynamic changes in rare or morphologically ambiguous cell populations such as pericytes. Flow cytometry (FACS), being high-throughput and quantitative, allows precise measurement of marker expression in defined cell populations (Crouch and Doetsch, 2018).

In this study, we investigated the therapeutic effects and cellular responses induced by immediate postreperfusion administration of PDGF-B HSPNPs in a tMCAO mice model. We hypothesized that early delivery of PDGF-B HSPNPs would enhance pericyte activation—reflected by upregulation of CD13 and PDGFRβ expression—and thereby contribute to improved neuroprotection and functional recovery after acute cerebral infarction. In addition, we introduce a novel flow cytometric approach to quantitatively assess activated pericyte populations at an early poststroke time point.

## Materials and Methods

### Purification of HSPNPs and synthesis of PDGF-B HSPNPs

The HSPG41C mutant encoded by the pET21a (1) vector was generated by polymerase chain reaction mutagenesis (Toita et al., 2012). HSPG41C mutant protein was purified from *E. coli* strain BL21 (DE3; Merck) using anion-exchange and size-exclusion chromatography (SEC). *E. coli* containing the pET21a (1) plasmid vector were cultured and induced with isopropyl-β-d-thiogalactopyranoside (Wako Pure Chemical Industries) to express the recombinant protein. After harvesting by centrifugation, cells were suspended in monopotassium phosphate solution containing EDTA and dithiothreitol, followed by sonication to disrupt cell membranes. The cell lysate was centrifuged, and the supernatant was collected. Proteins were purified using a HiPrep QHP 16/10 anion-exchange column (GE Healthcare) and an SEC column (TSKgel G4000SW; Tosoh).

HSPNPs were modified via amide bond formation with isothiocyanobenzyl-NTA (*N*-[5-(4-isothiocyanatobenzyl) amido-1-carboxypentyl] iminodiacetic acid; Dojindo Laboratories) at a 20-fold molar excess per protein monomer in HEPES (0.1 M), pH 8.0. Unreacted isothiocyanobenzyl-NTA was removed by ultrafiltration. For PDGF-B modification, recombinant human PDGF-B protein with a His tag (SPEED BioSystems) was prepared. HSPNP-modified-NTA and PDGF-B protein (two equivalents per HSPG41C) were dissolved in HEPES buffer (0.1 M), pH 8.0, containing nickel (II) and stirred for 24 h at 4°C, followed by ultrafiltration with a molecular weight cutoff of 100,000 to remove unreacted protein.

### Mouse stroke model

All animal experiments complied with the Animal Research: Reporting In Vivo Experiments guidelines (Percie du Sert et al., 2020). All animal procedures were performed in accordance with the authors’ university animal care committee’s regulations. Animals were randomly assigned to experimental groups prior to the surgical procedure. All outcome assessments and data analyses were performed by investigators blinded to group allocation. CB-17 (CB-17/lcr-1/1Jcl) wild-type mice were purchased from CLEA Japan. A total of 138 male and female mice, aged 8–24 weeks and weighing 20–33 g, were used. Animals were housed under a 12 h light/12 h dark cycle (LD 12:12). The onset of the light phase was designated as zeitgeber time (ZT) 0, and the onset of the dark phase as ZT 12. All animal experiments were conducted during the light phase. All mice were housed individually. The tMCAO model was generated as previously described (Kasahara et al., 2013). CB-17 mice were selected for their limited collateral circulation, which minimizes variability in infarct volume and allows clear evaluation of nanoparticle effects. CB-17 mice were established from the BALB/c strain and are characterized by a poor dural collateral circulation, reported to provide high reproducibility in creating ischemic areas (Taguchi et al., 2010; Tachibana et al., 2017). The 2 h occlusion period was selected based on established protocols demonstrating reliable and reproducible infarct formation in CB-17 mice while avoiding excessive mortality. This duration allows for consistent induction of ischemic injury and has been widely used in experimental stroke models (Kasahara et al., 2013).

Mice were randomly assigned to either the surgery or sham surgery groups. Anesthesia was induced by intraperitoneal injection of ketamine (100 mg/kg) and xylazine (10 mg/kg). Rectal temperature was maintained at 37°C with a heating pad. With the mouse in the lateral recumbent position, a skin incision was made along the midline between the left orbit and external auditory canal. After dissection of the subcutaneous tissue and temporalis muscle, the temporal bone was exposed. A burr hole was drilled using a microdrill to expose the middle cerebral artery (MCA). A 6-0 nylon suture was threaded distal to the olfactory tract under the MCA. The suture was rotated 180° clockwise, completely blocking blood flow to the MCA. Mice were returned to cages and maintained at 35–37°C until reperfusion. After 2 h of occlusion, mice were reanesthetized, sutures were removed by counterclockwise rotation, and the MCA was reperfused. PDGF-B HSPNPs or HSPNPs were administered intravenously via the tail vein immediately after reperfusion.

All evaluations were performed 3 d after nanoparticle administration. The intervention group received PDGF-B HSPNPs (PDGF-B HSPNPs group), while the control group received unmodified HSPNPs (HSPNPs group).

**Figure 1.**
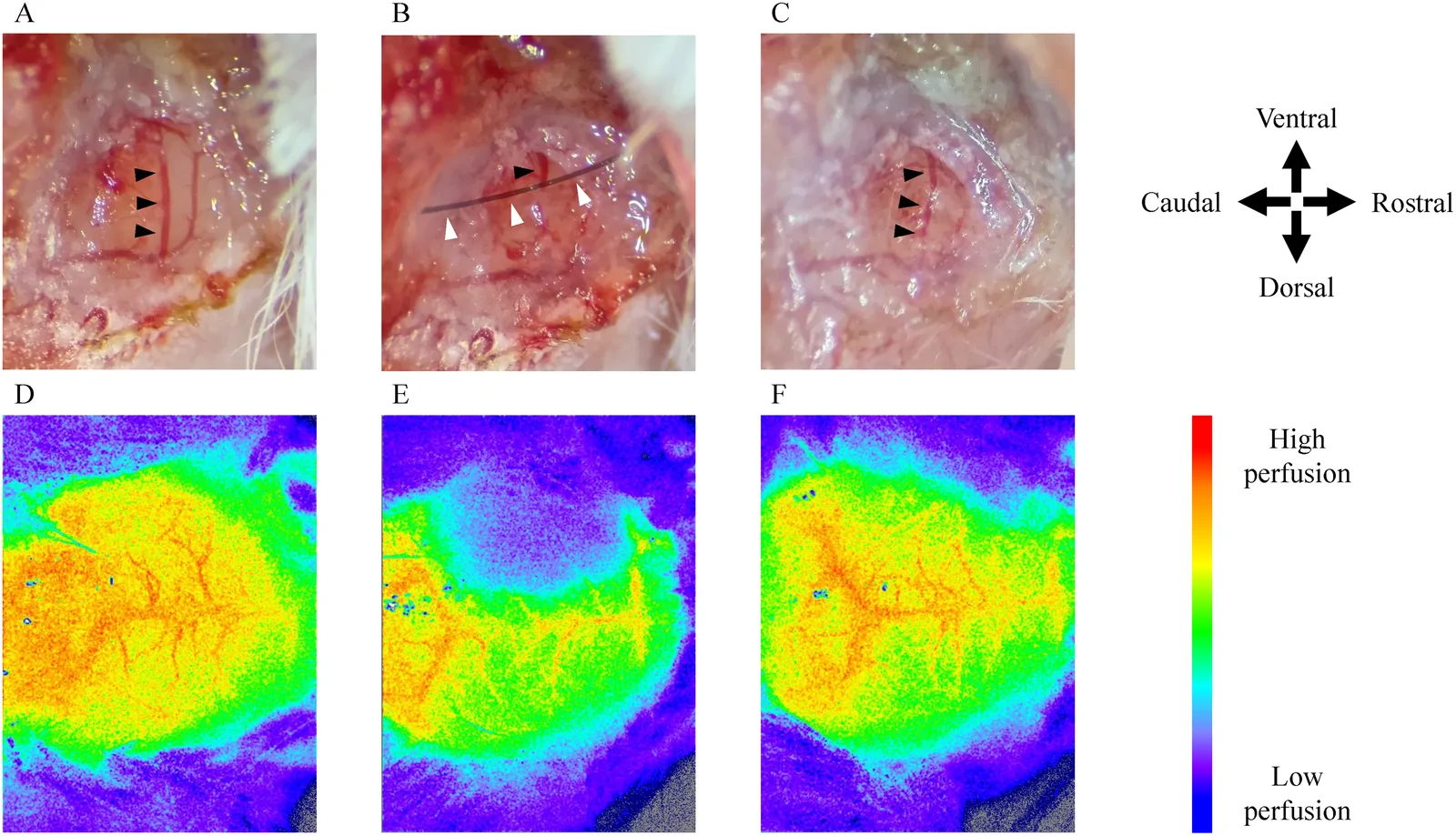
Verification of cerebral occlusion and reperfusion. ***A–C***, Direct microscopic confirmation of blood flow changes during MCA occlusion. ***A***, Baseline before occlusion. ***B***, Interruption of arterial blood flow during occlusion. ***C***, Restoration of blood flow after suture withdrawal (reperfusion). The MCA is indicated by black arrowheads, and the suture is indicated by white arrowheads. Interruption of blood flow by the suture is clearly visible during occlusion and reperfusion of flow is confirmed after suture withdrawal. Orientation: ventral is up, dorsal is down, caudal is left, and rostral is right. The mouse is positioned in a right lateral recumbent position on the surgical platform, with the left side of the skull exposed for the procedure. ***D–F***, Two-dimensional laser blood flow imaging of cortical perfusion analyzed using Omegazone OZ-2. ***D***, Baseline perfusion before occlusion. ***E***, Markedly reduced cortical perfusion during occlusion. ***F***, Recovery of cortical perfusion after reperfusion. Cortical blood flow is displayed in perfusion units (PU) using a pseudocolor scale ranging from blue (low perfusion) to red (high perfusion). The image was rotated to match the orientation of the surgical procedure figures. Orientation: the left hemisphere is shown at the top, the right hemisphere is shown at the bottom, caudal is left, and rostral is right.

Occlusion and reperfusion were confirmed both visually and by perfusion imaging. Under an operating microscope, interruption of arterial blood flow during tMCAO and restoration of flow after suture withdrawal were directly observed (Fig. 1*A*–*C*). In addition, cortical perfusion was assessed using two-dimensional laser blood flow imaging analyzed with Omegazone OZ-2 (Omegawave), demonstrating a marked reduction in perfusion during occlusion and recovery after reperfusion (Fig. 1*D*–*F*).

### Evaluation of nanoparticle accumulation in the infarct area

Accumulation of nanoparticles in the infarcted area was confirmed using fluorescently labeled nanoparticles and an in vivo imaging system (IVIS; PerkinElmer). HSPNPs and PDGF-B HSPNPs were fluorescently labeled with Alexa Fluor 488-maleimide (1 equivalent per protein monomer; Invitrogen) in 0.1 M phosphate buffer, pH 7.2, at 4°C for 24 h. Unreacted dye was removed by ultrafiltration and spin column purification. Labeling was performed via Michael addition of maleimide to thiol groups, forming stable bonds under physiological conditions. Labeled HSPNPs or PDGF-B HSPNPs (200 ml, 5 nmol) were injected via the tail vein immediately after reperfusion. Three days after the injection, mice were perfused transcardially with 1× HBSS, and fluorescence was measured using IVIS.

### In vivo magnetic resonance imaging (MRI)

MRI was performed using a 9.4 T Biospec scanner (Bruker). Mice were anesthetized as in the cerebral infarction model, fixed in an MR holder, and placed in a 3.8-cm-diameter birdcage coil. T2-weighted TurboRARE (Turbo Rapid Acquisition with Relaxation Enhancement, Bruker) images were acquired 3 d after tMCAO. Infarct volume was quantified using an image analysis system (NIH ImageJ; repetition time, 3,000 ms; echo time, 33 ms; number of signals averaged, 8; matrix resolution, 128 × 80; slice thickness, 1 mm; interslice distance, 1 mm; number of slices, 20; Wild et al., 2000). Infarct volume was quantified using an image analysis system (NIH ImageJ). The formula for calculating infarct volume is shown below:

Infarctvolumeratio=(ipsilateralT2highintensityvolume)/(contralateralhemispherevolume×2).

### Cylinder test and corner turn test

Behavioral assessment was conducted using the cylinder test (Li et al., 2004) and the corner turn test (Zhang et al., 2002), suitable for detecting motor deficits. Cylinder tests and the corner turn tests were performed on Day 3 after surgery.

In the cylinder test, mice were placed in acrylic cylinders (9 cm diameter, 20 cm height) and two mirrors positioned behind the cylinder facilitated observation of forelimb contacts with the wall. The use of both forelimbs was recorded as “both.” If the right forelimb slipped after simultaneous use of both forelimbs, it was recorded as “both” and “left.” A total of 20 trials were recorded per mouse. The formula for calculating cylinder test score is shown below:

Cylindertestscore=(leftforelimbexercise–rightforelimbexercise)/(leftforelimbexercise+rightforelimbexercise+bothexercises).

The score was 0 under normal conditions.

In the corner turn test, mice were placed in a corner with a 30° angle between two boards. When the mice reached the corner, the direction of turning (left or right) after exploratory rearing was recorded. The test was repeated 20 times with an intertrial interval of at least 30 s. In this study, turning to the left side was defined as an ipsilateral turn. The percentage of ipsilateral turns was calculated as (number of left turns/20) × 100%. Turns made without exploratory rearing were excluded from the analysis.

### Evaluation of the infarct area by microtubule-associated protein 2 (MAP2) staining

Mice were perfused transcardially with 1× HBSS before immunohistochemistry. Brains were sectioned using a mouse brain slicer (Muromachi), and 2-mm-thick coronal slices were prepared. Paraformaldehyde-fixed slices were embedded in paraffin and sectioned at 4 µm thickness. Sections were deparaffinized, and antigen retrieval was performed by microwaving in 10 mmol/L sodium citrate and 10 mmol/L trisodium citrate dehydrate buffer for 20 min. After washing with distilled water, endogenous peroxidase was blocked with 0.3% hydrogen peroxide-methanol solution for 30 min. Sections were then blocked with Dako REAL Antibody Diluent (Dako) containing 5% skim milk for 30 min at room temperature and incubated overnight at 4°C with anti-MAP2 antibody (1:100, clone: H-300; Santa Cruz Biotechnology). After MAP2 antibody reaction, the sections were washed with phosphate-buffered saline (PBS) containing 0.1% Triton X-100. Two drops of Histofine simple stain MAX PO anti-mouse immuno-peroxidase polymer (Nichirei Biosciences) were dropped on top of the specimen and allowed to react for 30 min at room temperature. One drop of Histofine simple stain DAB solution (Nichirei Biosciences) was then dropped onto the sections and allowed to react for 5 min. Infarct lesions were defined as MAP2-negative areas under a microscope. Infarct volume was quantified using an image analysis system (NIH ImageJ). The formula for calculating infarct volume is shown below:

MAP2negativeratio=(ipsilateralMAP2negativearea)/(contralateralhemisphere×2).

Infarct volume analysis using MAP-2 staining was performed in a separate cohort of animals and was not expanded in conjunction with the repeated behavioral experiments.

### Immunohistochemistry of cell markers

Sections were deparaffinized and subjected to antigen retrieval by autoclaving in 10 mmol/L sodium citrate and 10 mmol/L trisodium citrate dihydrate buffer for 20 min. After washing with distilled water, sections were blocked with Dako REAL Antibody Diluent (Dako) containing 5% skim milk for 30 min at room temperature. CD13 antibody (1:200; clone, EPR4058; Abcam), PDGFRβ antibody (1:200; clone, APB5; Novus Biologicals), and CD31 antibody (1:200; clone, MEC13.3; Novus Biologicals) were applied and incubated overnight at 4°C.

After washing with PBS containing 0.1% Triton X-100, sections were incubated with Alexa Fluor-conjugated secondary antibodies for 30 min at room temperature. After washing the sections with PBS containing 0.1% Triton X-100, each antibody expression was evaluated microscopically. The positive area was quantified using an image analysis system (NIH ImageJ).

### Evaluation of pericytes using flow cytometry

Flow cytometry was performed to evaluate pericyte activity, following established protocols (Crouch and Doetsch, 2018) with modifications described below. Brain samples were collected from mice after transcardial perfusion with 1× HBSS, placed in culture medium, and minced into small pieces. The tissue was centrifuged and enzymatically dissociated in Nuclease-free Water/PBS containing collagenase/dispase (Roche, catalog #11097113001; final concentration 100 mg/ml) at 37°C for 30 min with gentle agitation. To prevent cell aggregation, DNase I (Worthington Biochemical, catalog #LS002139; final concentration 10 mg/ml) was added during digestion with pipetting 100 times. The tissue was subjected to a 22% Percoll density gradient to enrich viable cells. Cells were washed and centrifuged in HBSS containing BSA and glucose. For immunostaining, cells were incubated with anti-CD16/32 antibody to block Fc receptor–mediated nonspecific binding. Dead cells were excluded using 7-aminoactinomycin D (7-AAD), which was incubated together with CD16/32 for 10 min, followed by quenching with EDTA-containing buffer. Cells were then incubated for 12 min with antibodies against CD13 (pericyte marker), CD140b (PDGFRβ; marker of activated pericytes), CD31 (endothelial marker), CD41 (to exclude platelets), and CD45 (to exclude leukocytes). Antibodies used are listed in Table 1. After staining, cells were washed with staining buffer composed of PBS (500 ml) supplemented with EDTA (2 ml) and FBS (2.5 ml) and passed through cell strainers immediately prior to acquisition to obtain a single-cell suspension. Samples were then analyzed immediately. Flow cytometry was performed using a BD FACSLyric system, acquiring 100,000 events per sample. Data were analyzed using the FlowJo software (version 10.5.3; BD Biosciences). Gating was performed sequentially to exclude debris, doublets, and dead cells (7-AAD^+^), followed by exclusion of hematopoietic and platelet populations (CD45^+^ and CD41^+^). Pericytes were defined as CD13^+^ cells, endothelial cells were defined as CD31^+^ cells, and activated pericytes were identified as the CD13^+^/PDGFRβ^+^ subset. Fluorescence minus one (FMO) controls were used to set gates for all markers.

**Table 1.** Information on primary antibodies used in flow cytometry

Antibodies	Source	Dilutions	Manufacturer	Clone
CD13	Rat	1:50	BD Pharmingen	R3–242
CD140b	Rat	1:100	BioLegend	APB5
CD31	Rat	1:50	BioLegend	MEC13.3
CD41	Rat	1:200	BioLegend	MWReg30
CD45	Rat	1:200	BioLegend	30-F11

Source, dilutions, manufacturer, and clone of the antibodies used in flow cytometry.

### Statistical analysis

Data are presented as mean ± standard deviation. Statistical analyses were performed using unpaired *t* tests in the JMP software (version 18; SAS Institute). A *p* value of <0.05 was considered statistically significant. Statistical table is shown in Table 2.

**Table 2.** Statistical table

Evaluation items	Group	Data structure	Type of test	Confidence intervals
Infarct volume ratio				
	HSPNPs	Normal distribution	*t* test (2-tailed)	0.11475, 0.13699
	PDGF-B HSPNPs	Normal distribution	*t* test (2-tailed)	0.09368, 0.11592
MAP-2 negative ratio				
	HSPNPs	Normal distribution	*t* test (2-tailed)	0.10779, 0.14584
	PDGF-B HSPNPs	Normal distribution	*t* test (2-tailed)	0.07278, 0.10574
Cylinder test score				
	HSPNPs	Normal distribution	*t* test (2-tailed)	0.22511, 0.34489
	PDGF-B HSPNPs	Normal distribution	*t* test (2-tailed)	0.36011, 0.47989
Percentage (%) of ipsilateral (left) turns of the corner turn test				
	HSPNPs	Normal distribution	*t* test (2-tailed)	81.306, 87.266
	PDGF-B HSPNPs	Normal distribution	*t* test (2-tailed)	75.591, 81.552
CD13 positive (frequency of live cells)				
	HSPNPs	Normal distribution	*t* test (2-tailed)	0.02739, 0.09011
	PDGF-B HSPNPs	Normal distribution	*t* test (2-tailed)	0.09056, 0.15328
CD13 positive (absolute cell number)				
	HSPNPs	Normal distribution	*t* test (2-tailed)	2,918, 11,104
	PDGF-B HSPNPs	Normal distribution	*t* test (2-tailed)	11,328, 19,514
CD140b positive (frequency of live cells)				
	HSPNPs	Normal distribution	*t* test (2-tailed)	0.01525, 0.06781
	PDGF-B HSPNPs	Normal distribution	*t* test (2-tailed)	0.07066, 0.12322
CD140b positive (absolute cell number)				
	HSPNPs	Normal distribution	*t* test (2-tailed)	1,745.2, 8,146
	PDGF-B HSPNPs	Normal distribution	*t* test (2-tailed)	9,001.8, 15,402
CD31 positive (frequency of live cells)				
	HSPNPs	Normal distribution	*t* test (2-tailed)	0.00981, 0.06246
	PDGF-B HSPNPs	Normal distribution	*t* test (2-tailed)	0.04879, 0.10145
CD31 positive (absolute cell number)				
	HSPNPs	Normal distribution	*t* test (2-tailed)	1,178.2, 7,441
	PDGF-B HSPNPs	Normal distribution	*t* test (2-tailed)	6,519.0, 12,782
CD13 CD140b double positive (frequency of live cells)				
	HSPNPs	Normal distribution	*t* test (2-tailed)	0.01384, 0.06632
	PDGF-B HSPNPs	Normal distribution	*t* test (2-tailed)	0.06541, 0.11790
CD13 CD140b double positive (absolute cell number)				
	HSPNPs	Normal distribution	*t* test (2-tailed)	1,540.4, 8,004
	PDGF-B HSPNPs	Normal distribution	*t* test (2-tailed)	8,313.8, 14,778
CD13 positive area in the peri-infarct area				
	HSPNPs	Normal distribution	*t* test (2-tailed)	0.1638, 1.1033
	PDGF-B HSPNPs	Normal distribution	*t* test (2-tailed)	3.0270, 3.9665
PDGFRβ-positive area in the peri-infarct area				
	HSPNPs	Normal distribution	*t* test (2-tailed)	0.07816, 0.15056
	PDGF-B HSPNPs	Normal distribution	*t* test (2-tailed)	0.16124, 0.23364
CD13 positive area in the peri-infarct area				
	HSPNPs	Normal distribution	*t* test (2-tailed)	0.2347, 0.9819
	PDGF-B HSPNPs	Normal distribution	*t* test (2-tailed)	2.0612, 2.8084
CD13 positive area in the intrainfarct area				
	HSPNPs	Normal distribution	*t* test (2-tailed)	0.19664, 0.82280
	PDGF-B HSPNPs	Normal distribution	*t* test (2-tailed)	0.32032, 0.94648
PDGFRβ-positive area in the intrainfarct area				
	HSPNPs	Normal distribution	*t* test (2-tailed)	0.04583, 0.10857
	PDGF-B HSPNPs	Normal distribution	*t* test (2-tailed)	0.05595, 0.11869
CD13 positive area in the intrainfarct area				
	HSPNPs	Normal distribution	*t* test (2-tailed)	0.02972, 0.43053
	PDGF-B HSPNPs	Normal distribution	*t* test (2-tailed)	0.20539, 0.60620

Types of tests and measures of confidence intervals used.

## Results

### PDGF-B HSPNPs accumulation in the infarct area

**Figure 2.**
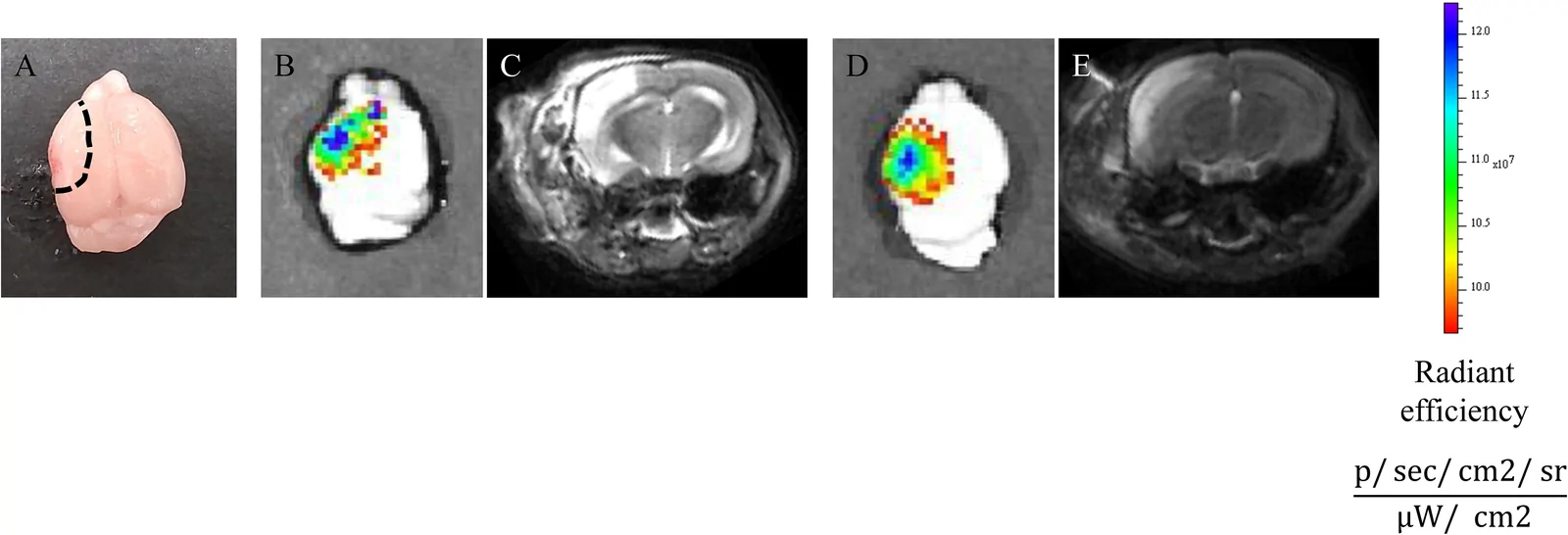
Accumulation of HSPNPs in the infarcted region. Fluorescently labeled HSPNPs and PDGF-B HSPNPs (200 µl, 5 nmol) injected via the tail vein immediately after recanalization in tMCAO mice were evaluated using IVIS and MRI. ***A***, Dorsal photograph of the mouse brain. Dotted lines indicate the infarcted area. ***B***, HSPNPs. ***C***, PDGF-B HSPNPs.

To examine the accumulation of HSPNPs or PDGF-B HSPNPs in the infarct area, we analyzed the distribution of fluorescently labeled HSPNPs or PDGF-B HSPNPs (200 ml, 5 nmol) in tMCAO mice using MRI and IVIS. Fluorescently labeled nanoparticles were injected immediately after reperfusion, and nanoparticle accumulation was assessed on Day 3. Accumulation of both HSPNPs and PDGF-B HSPNPs was clearly observed in the infarct areas (Fig. 2). Serum AST, ALT, and creatinine levels measured before and 3 d after nanoparticle administration remained within the normal physiological range, without overt abnormalities or unexpected deaths (Extended Data Fig. 2-1).

10.1523/ENEURO.0404-25.2026.f2-1 Figure 2-1 **Assessment of hepatic and renal toxicity following intravenous administration of PDGF-B HSPNPs in CB-17 mice** Serum biochemical parameters in untreated CB-17 mice (Pre-injection) and 3 days after intravenous administration of PBS (Control) or PDGF-B HSPNPs. Mice received 200 μL PBS or PDGF-B HSPNPs via tail vein injection and were euthanized 3 days later for blood collection. Serum AST, ALT, and creatinine levels were measured to assess systemic toxicity. Download Figure 2-1, TIF file.

### PDGF-B HSPNPs reduced infarct volume and improved motor function

**Figure 3.**
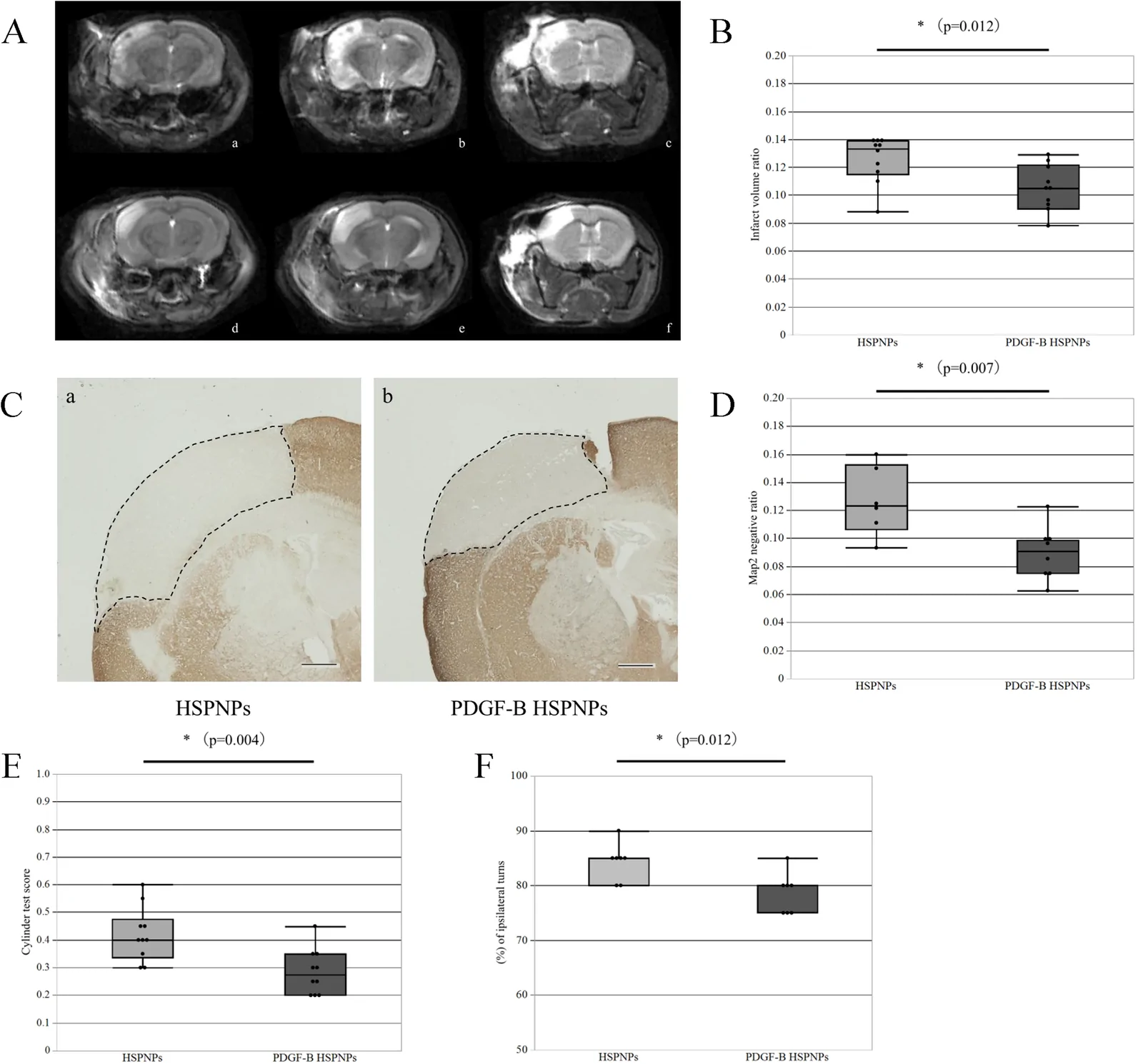
Therapeutic effect of PDGF-B HSPNPs on infarct volume and motor function after tMCAO. Infarct volume was evaluated 3 d after administration of HSPNPs or PDGF-B HSPNPs using both MRI and MAP-2 immunohistochemistry. ***A***, Representative MRI results obtained 3 d after treatment: top panels (***a–c***), HSPNPs group; bottom panels (***d–f***), PDGF-B HSPNPs group. ***B***, Infarct volume ratio was significantly lower in the PDGF-B HSPNP group compared with the HSPNP group. Values are mean ± SD (*N* = 10 per group; *p < 0.05; n.s., not significant). Infarct volume was calculated using the following formula: infarct volume ratio = (ipsilateral T2 high intensity area)/(contralateral hemisphere × 2). ***C***, Representative MAP-2 staining images 3 d after treatment. ***a***, HSPNPs. ***b***, PDGF-B HSPNPs (scale bars, 500 µm). Infarct areas are indicated by dotted areas. ***D***, The MAP-2 negative ratio was significantly lower in the PDGF-B HSPNP group compared with the HSPNP group. Values are mean ± SD (*N* = 6 for HSPNPs; *N* = 8 for PDGF-B HSPNPs; *p < 0.01; n.s., not significant). MAP-2-negative ratio = (ipsilateral MAP-2-negative area)/(contralateral hemispheric area × 2). ***E, F***, Motor function was evaluated using (***E***) the cylinder test and (***F***) the corner turn test on Day 3 in the HSPNP and PDGF-B HSPNP group. Cylinder test score = (left forelimb exercise − right forelimb exercise)/(left forelimb exercise + right forelimb exercise + both exercises). A score of 0 indicates no motor paralysis. Values are mean ± SD (*N* = 10 per group; *p < 0.01; n.s., not significant). The percentage (%) of ipsilateral turns = (left turns/20) × 100%. Higher values indicate greater turning toward the ipsilateral (paretic) side. Values are mean ± SD (*N* = 7 per group; *p < 0.05; n.s., not significant).

We next investigated the therapeutic effect of PDGF-B HSPNPs in tMCAO mice by analyzing infarct volume and motor function. MRI (T2-weighted imaging) demonstrated that intravenous administration of PDGF-B HSPNPs immediately after MCA recanalization significantly reduced infarct volume compared with HSPNPs (Fig. 3*A*,*B*). Consistent with the MRI results, immunohistochemistry indicated that infarct volume assessed by MAP-2 staining was significantly reduced on Day 3 in the PDGF-B HSPNPs group compared with the HSPNPs group (Fig. 3*C*,*D*). This analysis was included as a supportive histological endpoint rather than a primary outcome measure and should be interpreted accordingly.

We then evaluated functional recovery using the cylinder test and the corner turn test. Motor function assessed on Day 3 was significantly improved in the PDGF-B HSPNPs group compared with the HSPNPs group (Fig. 3*E*,*F*). These findings suggest that intravenous administration of PDGF-B HSPNPs immediately after reperfusion decreased infarct volume and improved motor recovery in tMCAO mice.

### PDGF-B HSPNPs enhanced pericyte activation

**Figure 4.**
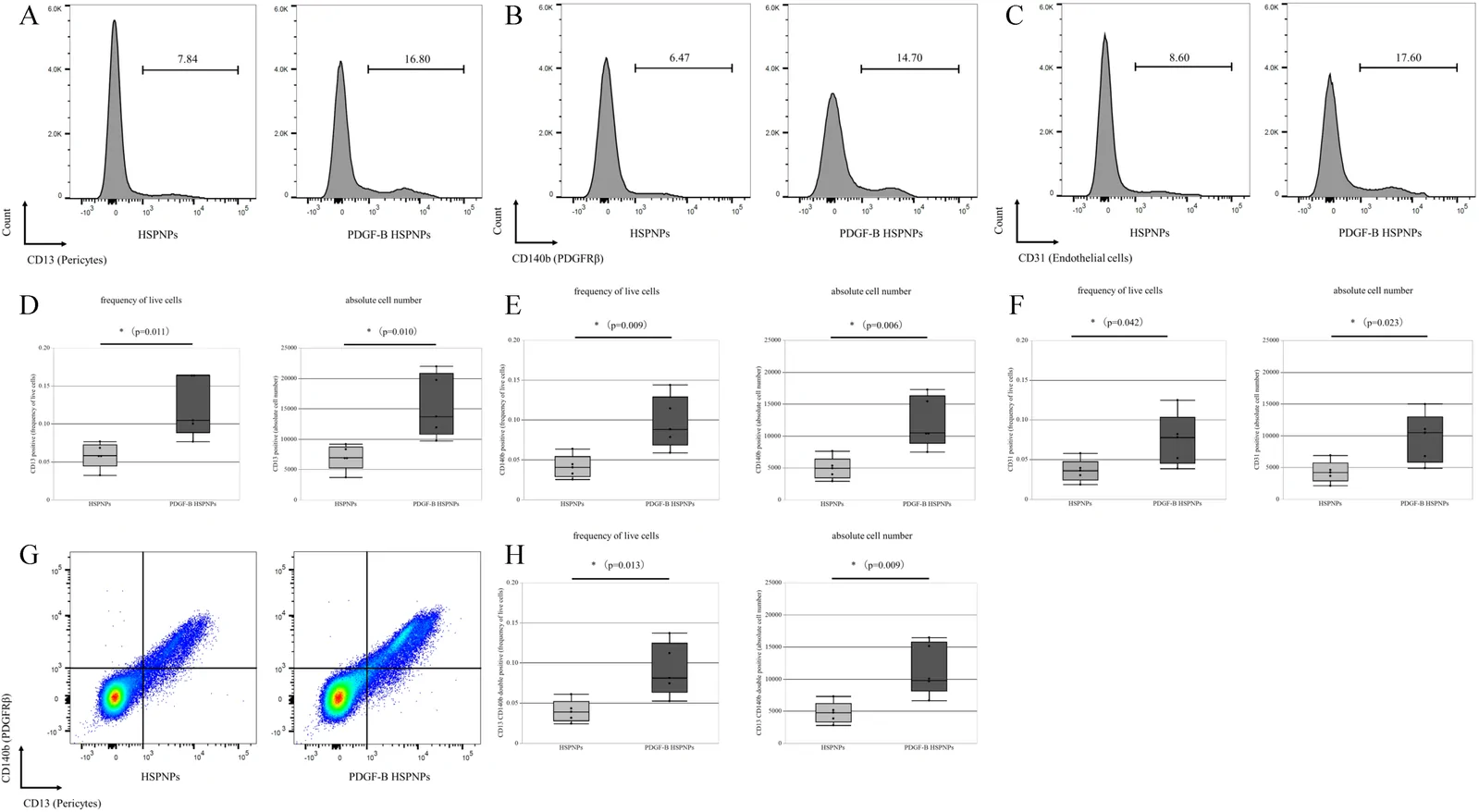
Evaluation of activated pericytes and endothelial cells following PDGF-B HSPNP treatment using flow cytometry. ***A–C***, Flow cytometry showing histograms of (***A***) CD13 (+) cells, (***B***) CD140b (+) cells, and (***C***) CD31 (+) cells. The numbers indicate the frequency of positive cells. ***D–F***, Frequency of live cells and absolute numbers of (***D***) CD13 (+) cells, (***E***) CD140b (+) cells, and (***F***) CD31 (+) cells at 3 d after administration of HSPNPs and PDGF-B HSPNPs. ***G***, Multicolor panel with CD13 on the horizontal axis and CD140b on the vertical axis: the top right quadrant represents CD13^+^CD140b^+^ cells, indicating activated pericytes. Plots show 100,000 events. ***H***, Frequency of live cells and absolute cell number of CD13 (+) CD140b (+) cells at 3 d after administration of HSPNPs or PDGF-B HSPNPs. FSC-A; forward-scatter area. Values are mean ± SD (*N* = 5 per group; *p < 0.05; n.s., not significant).

To further investigate the mechanisms underlying the therapeutic effect of PDGF-B HSPNPs, we performed flow cytometry on mouse brain samples to assess pericyte activation. Multicolor flow cytometry plots indicated increased populations of live cells expressing CD13 (pericyte marker), CD140b (PDGFRβ marker), and CD31 (endothelial marker) in the PDGF-B HSPNPs group compared with the HSPNPs group (Fig. 4*A*–*C*). Similarly, the frequency and absolute numbers of CD13(+), CD140b(+), and CD31(+) cells were significantly higher in the PDGF-B HSPNPs group than in the HSPNPs group (Fig. 4*D*–*F*). These results indicate that PDGF-B HSPNPs promoted an increase in both pericytes and PDGFRβ-expressing cells, as well as endothelial cells.

Furthermore, the proportion and absolute number of CD13 CD140b double positive cells, representing activated pericytes, were significantly higher in the PDGF-B HSPNPs group compared with the HSPNPs group (Fig. 4*G*,*H*). These findings suggest that PDGF-B HSPNPs increased the number of activated pericytes expressing PDGFRβ.

Representative gating plots (Extended Data Fig. 4-1) and FMO controls for the multicolor panel (Extended Data Fig. 4-2*A–F*) are shown to validate the specificity of the staining and the gating strategy used for quantification.

10.1523/ENEURO.0404-25.2026.f4-1 Figure 4-1 **Representative gating plots for the multicolor panel flow cytometry of mouse brain.** Single-cell suspensions from adult mouse brain were analyzed by flow cytometry as described in Methods. Forward scatter area (FSC-A) versus side scatter area (SSC-A) plot used to exclude debris. Singlets were gated using Forward scatter area (FSC-A) versus Forward scatter height (FSC-H) to remove doublets. Live cells were identified by exclusion of 7-AAD⁺ dead cells. Hematopoietic and platelet populations were excluded by gating out CD41⁺ and CD45⁺ cells. Download Figure 4-1, TIF file.

10.1523/ENEURO.0404-25.2026.f4-2 Figure 4-2 **Representative fluorescence-minus-one (FMO) controls for the multicolor panel flow cytometry of mouse brain.** Representative fluorescence-minus-one (FMO) control excluding A: FITC-conjugated anti-CD13 antibody, B: PE-conjugated anti-CD31 antibody, C: APC-conjugated anti-CD140b antibody, D: Brilliant Violet 605-conjugated anti-CD41 antibody, E: APC/Cyanine7-conjugated anti-CD45 antibody, F: 7-AAD Download Figure 4-2, TIF file.

### Immunohistochemical analysis of activated pericytes after PDGF-B HSPNP treatment

**Figure 5.**
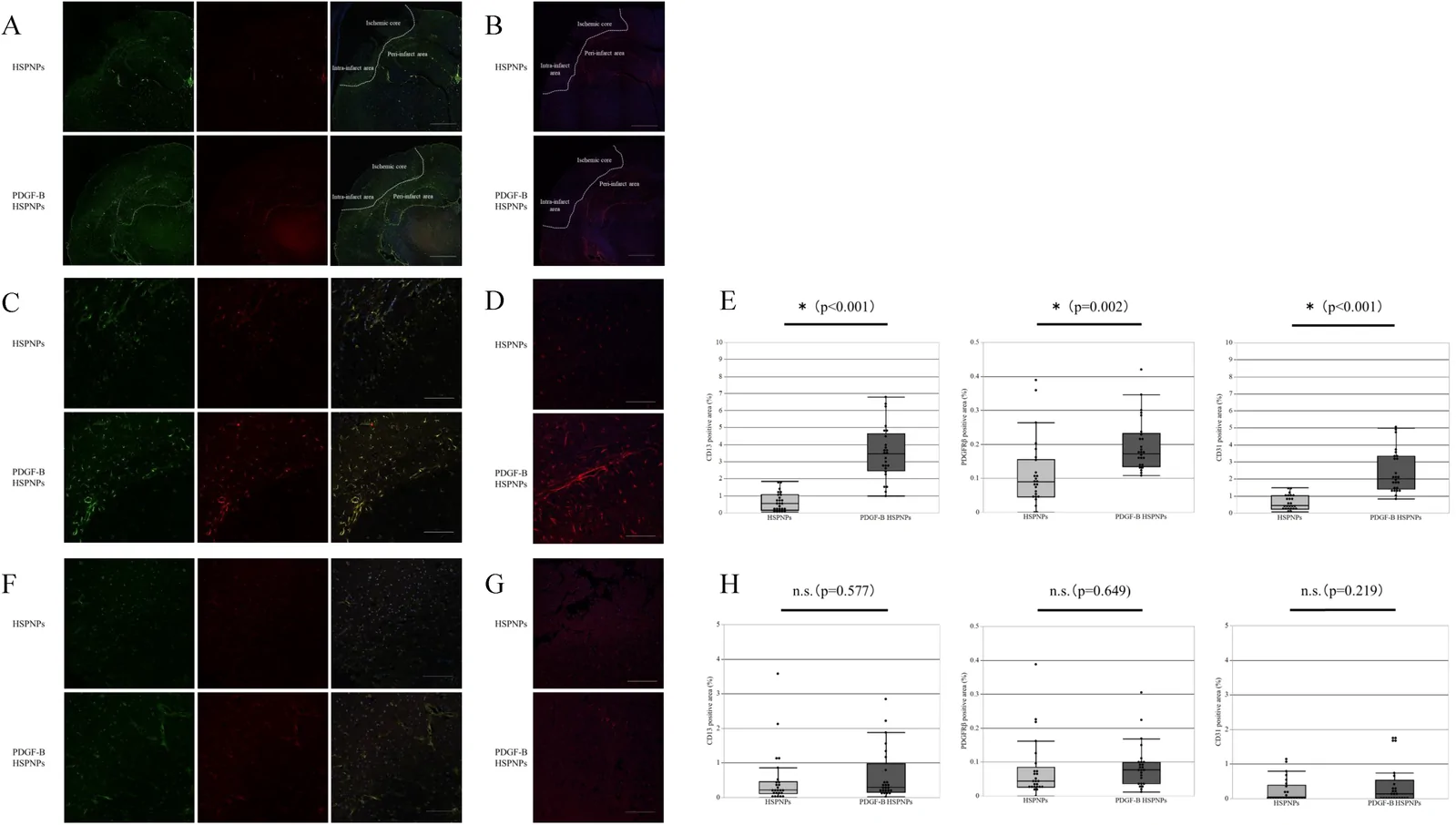
Distribution of activated pericytes in the infarct region. ***A***, Immunohistochemistry for CD13 (green), PDGFRβ (red), and merged images with Hoechst nuclear staining (blue) performed 3 d after administration of HSPNPs and PDGF-B HSPNPs. Top panels, HSPNS group; bottom panels, PDGF-B HSPNPs group. Scale bar, 1,000 µm. ***B***, Immunohistochemistry for CD31 (red) with Hoechst nuclear staining (blue) performed 3 d after administration of HSPNPs and PDGF-B HSPNPs. Top panels, HSPNS group; bottom panels, PDGF-B HSPNPs group. Scale bar, 1,000 µm. ***C, D***, Higher-magnification views of the peri-infarct area (40×). ***C***, Representative image for CD13 (green), PDGFRβ (red), and merged images with Hoechst nuclear staining (blue). ***D***, Representative image for CD31 (red) with Hoechst nuclear staining (blue). Scale bar, 100 µm. ***E***, Quantification of CD13 (+), PDGFRβ (+), and CD31 (+) areas in the peri-infarct area (*N* = 5 per group, five fields per sample). ***F, G***, Higher-magnification views of the intrainfarct area (40×). ***F***, Representative image for CD13 (green), PDGFRβ (red), and merged images with Hoechst nuclear staining (blue). ***G***, Representative image for CD31 (red) with Hoechst nuclear staining (blue). Scale bar, 100 µm. ***H***, Quantification of CD13 (+), PDGFRβ (+), and CD31 (+) areas in the intrainfarct area (*N* = 5 per group, five fields per sample).

Finally, immunohistochemical staining using anti-CD13, anti-PDGFRβ, and anti-CD31 antibodies was performed to confirm the distribution of activated pericytes in the intrainfarct area and peri-infarct area after HSPNPs and PDGF-B HSPNP treatment. CD13(+), PDGFRβ (+), and CD31(+) cells were significantly increased in the peri-infarct area on Day 3 after PDGF-B HSPNP administration compared with HSPNPs, whereas no significant difference was observed in the intrainfarct area (Fig. 5*A*–*H*). These findings suggest that PDGF-B HSPNPs promote the accumulation of CD13(+) PDGFRβ(+) cells, representing activated pericytes, primarily in the peri-infarct area compared with HSPNP administration, along with an increase in CD31(+) endothelial area in this region.

## Discussion

In this study, we demonstrated that intravenous administration of PDGF-B HSPNPs immediately after MCA recanalization reduced infarct volume and improved motor function recovery in tMCAO mice compared with unmodified HSPNPs. Flow cytometry analysis revealed that PDGF-B HSPNPs increased CD13(+), CD140b(+), and CD31(+) cells relative to HSPNPs. Both the proportion and absolute number of CD13(+) CD140(+) cells were also higher after PDGF-B HSPNPs treatment compared with HSPNP treatment, suggesting an increase in activated pericytes. Immunohistochemical analysis further indicated that these activated pericytes were predominantly increased in the peri-infarct area rather than the intrainfarct area 3 d after PDGF-B HSPNP treatment. These findings suggest that administration of PDGF-B HSPNPs immediately after reperfusion may enhance pericyte activation in the peri-infarct area, potentially contributing to a neuroprotective role in transient cerebral ischemia.

In this study, PDGF-B HSPNPs were administered as a single intravenous injection immediately after reperfusion, and therapeutic efficacy was evaluated 3 d later. HSPNPs based on heat shock protein 16.5 are self-assembling nanoparticles that have been reported to maintain structural stability under serum-containing and in vivo conditions (Kawano et al., 2015, 2018; Murata et al., 2015). A limitation of the present study is that the blood half-life, biodistribution, tissue retention, and formal pharmacokinetic profile of PDGF-B HSPNPs were not directly evaluated. Therefore, the extent of nanoparticle exposure and accumulation in target tissues following administration cannot be quantitatively determined. Previous studies have reported that HSPNPs exhibit broad tissue distribution after intravenous administration, are largely cleared within 24 h, are primarily eliminated through urine and feces, and do not cause overt toxicity after a single injection in mice (Kaiser et al., 2007). Although dedicated toxicity studies were not performed in the present work, animals were routinely monitored throughout the experimental period and survived to the planned endpoint without overt clinical abnormalities. In addition, serum AST, ALT, and creatinine levels measured before and after nanoparticle administration (Supplementary Fig. 2-1) remained within the normal physiological range. Nevertheless, these observations do not constitute a comprehensive safety assessment. Future studies should include detailed pharmacokinetic, biodistribution, and toxicity analyses to define the in vivo behavior and safety profile of PDGF-B HSPNPs more precisely. Despite these limitations, the present findings suggest that a single administration of PDGF-B HSPNPs immediately after reperfusion can exert biologically meaningful effects during the acute postischemic phase, resulting in the neurovascular and functional benefits observed at 3 d after reperfusion.

Previous studies have demonstrated that various nanoparticle-based delivery systems can access the brain under ischemic conditions accompanied by BBB disruption. In contrast, for protein-based nanocage platforms, direct visualization of cellular uptake in the ischemic brain remains limited. In this context, the present study demonstrates that PDGF-B HSPNPs exert biologically meaningful functional effects on brain-resident cells in the ipsilateral cortex.

Pericytes are mesenchymal cells and a component of the vessel wall, residing at the level of capillaries to microvessels within the basement membrane surrounding endothelial cells (Kamouchi et al., 2011). Pericytes are considered part of the neurovascular unit (NVU) and are responsible for stabilizing the BBB by forming tight junctions through interaction with endothelial cells (Sweeney et al., 2016; Cheng et al., 2018; Schaeffer and Iadecola, 2021). The ratio of pericytes to endothelial cells varies considerably between organs, with skeletal muscle having a ratio of ∼1:100, while the central nervous system and retina exhibit the highest ratios (1:1 to 1:3) in vivo. The maintenance of pericyte function is therefore critical for BBB integrity and NVU health (Armulik et al., 2011; Zlokovic, 2011). During development, neurons secrete molecules such as vascular endothelial growth factor (VEGF) to induce angiogenesis, and endothelial cells form a lumen. Endothelial cells also secrete PDGF-B, which mobilizes PDGFRβ-expressing pericytes and triggers angiogenesis maturation. In PDGFRβ knock-out mice, pericytes fail to localize around endothelial cells, leading to abnormal vessel formation, cerebral hemorrhage, and death in the fetal or early postnatal period (Lindahl et al., 1997; Daneman et al., 2010). These results suggest that PDGF-B signaling is a key factor for pericyte activation.

Pericytes play multiple roles in neurological recovery after brain ischemia, including autoregulation of cerebral blood flow, maintenance of BBB, angiogenesis, neuroprotection via neurotrophic factor upregulation, multipotent stem cell activity, anti-inflammatory functions, and tissue repair (Caplan and Correa, 2011; Arimura et al., 2012; Shen et al., 2012; Makihara et al., 2015; Nakamura et al., 2016; Gaceb et al., 2018). We previously reported that recruitment of activated PDGFRβ-positive pericytes begin in the peri-infarct area during the acute phase after brain ischemia (Arimura et al., 2012; Makihara et al., 2015), suggesting that promoting recruitment of activated pericytes immediately after ischemia may represent a therapeutic target for brain infarction.

We also reported that intravenous administration of PDGF-B HSPNPs 1 d after tMCAO reduced infarct volume and improved functional recovery through antiapoptotic effects and enhanced tissue repair (Takagishi et al., 2021). These neuroprotective effects were considered to involve pericyte activation induced by PDGF-B HSPNPs, but the underlying mechanisms remained unclear. In the present study, flow cytometry analysis demonstrated that the numbers of CD13(+), CD140b(+), and CD31(+) cells were increased in the PDGF-B HSPNP group compared with the HSPNP group, suggesting that pericytes, PDGFRβ-positive cells, and endothelial cells were increased following PDGF-B HSPNP treatment. Furthermore, this work provides novel flow cytometric quantification of activated pericyte populations (CD13^+^CD140b^+^) at an early poststroke time point, offering new insight into pericyte dynamics during the acute phase of ischemic stroke. PDGF-B HSPNPs potentially contribute to the increase in pericytes and PDGFRβ-positive cells in the peri-infarct area via PDGF-B signaling. Previous studies have reported that pericytes contribute to endothelial cell sprouting and promote angiogenesis by inducing endothelial cells through the secretion of VEGF, Angiopoietin-1, and TGF-β (Eilken et al., 2017; Cao et al., 2021; Zhou et al., 2022). Taken together, these findings indicate that PDGF-B HSPNP treatment is associated with increased CD13 and PDGFRβ-positive perivascular cells and an increased CD31 positive area specifically within the peri-infarct area. These coordinated changes within components of the NVU may reflect modulation of microenvironment in the peri-infarct area, such as endothelial preservation or early vascular remodeling, which could contribute to the observed neuroprotective effects. However, as we did not directly assess vessel density, vascular architecture, or cerebral blood flow, the present data do not establish definitive evidence of angiogenesis.

Furthermore, immunohistochemical analysis showed that activated pericytes were predominantly increased in the peri-infarct area. In our previous study, we demonstrated that PDGFRβ-positive pericytes proliferate in the peri-infarct area after cerebral infarction and that in PDGFRβ heterozygous knock-out mice, this proliferation was reduced, impairing poststroke wound healing, leading to infarct expansion (Makihara et al., 2015). The present immunohistochemical findings suggest that PDGF-B HSPNPs promote pericyte activation in the peri-infarct area, enhancing multiple pericyte-mediated functions around the infarct and contributing to neuroprotection. Further studies are warranted to elucidate the downstream signaling pathways and the diverse pericyte functions enhanced by this treatment.

Finally, the PDGF-B HSPNP treatment used in this study—intravenous nanoparticle administration immediately after reperfusion therapy for cerebral infarction—is a minimally invasive and clinically applicable approach that could serve as a novel therapy for efficiently activating pericytes. Although issues remain regarding safety, detailed mechanisms, and efficacy in higher mammals, this strategy has the potential to become a new therapeutic option for neuroprotection after cerebral infarction.

This study has several limitations. First, it has not been conclusively proven that the neuroprotective effect of PDGF-B HSPNPs is mediated specifically through pericyte activation. Experiments using knock-out mice or pharmacological inhibitors will be required to clarify this point. Second, the precise localization of nanoparticles within the mouse brain has not been identified. Although PDGF-B HSPNP accumulation in the infarcted region was confirmed, it remains unclear whether they localize within blood vessels, near pericytes and endothelial cells, or within neuronal compartments. Evaluating the localization of PDGF-B HSPNPs will be necessary to elucidate the mechanism of action. Third, pharmacodynamic evaluation was lacking. To adapt nanoparticle-based drug delivery therapy for human stroke, it is essential to evaluate the dosage, blood concentration, and associations with clinical efficacy. This study did not assess clinically relevant conditions such as the timing of PDGF-B HSPNP administration, long-term therapeutic effects, efficacy in large stroke models, or evaluation in permanent occlusion models. Our goal is to conduct further research to develop novel stroke therapies using PDGF-B HSPNPs. Fourth, the effects of PDGF-B HSPNPs on the BBB were not directly evaluated in the present study. BBB disruption is known to occur rapidly after ischemia–reperfusion and is closely associated with pericyte and endothelial dysfunction. Although contrast-enhanced MRI would allow direct evaluation of BBB permeability, the present study focused on early cellular responses and pericyte activation during the acute phase. Future studies incorporating contrast-enhanced MRI will help clarify how PDGF-B HSPNPs influence BBB integrity and vascular remodeling. Fifth, this study was conducted exclusively in CB-17 mice, which are characterized by limited collateral circulation. While this choice reduced variability in infarct volume and improved reproducibility, it may limit the generalizability of our findings to other commonly used strains, such as C57BL/6, that exhibit different collateral profiles and patterns of penumbral evolution. Finally, behavioral assessments were limited to the acute phase within 3 d after reperfusion and included only the cylinder and corner turn tests, reflecting our focus on early neuroprotective effects of PDGF-B HSPNs. However, functional recovery after stroke evolves over time, and more comprehensive evaluation with multiple behavioral tests and extended time points (e.g., 7–14 d) would better capture long-term outcomes. Thus, our findings are restricted to acute functional improvements and do not address long-term recovery or neuroplasticity. Future studies will incorporate longer follow-up periods and broader behavioral assessments to address this limitation.

In conclusion, administration of PDGF-B HSPNPs immediately after MCA reperfusion reduced infarct volume and improved functional recovery in tMCAO mice, possibly through activation of pericytes in the peri-infarct area. These results suggest that PDGF-B HSPNPs may represent a promising therapeutic strategy for ischemic stroke. Further investigations are needed to confirm the underlying mechanisms and to enable clinical application.

## Synthesis

Reviewing Editor: Selva Baltan, Oregon Health &Science University Department of Surgery Decisions are customarily a result of the Reviewing Editor and the peer reviewers coming together and discussing their recommendations until a consensus is reached. When revisions are invited, a fact-based synthesis statement explaining their decision and outlining what is needed to prepare a revision will be listed below. The following reviewer(s) agreed to reveal their identity: Eduardo Candelario-Jalil.

Please follow the reviewers' comments carefully. The revised manuscript will be sent back to reviewers for their assessment.

The following points are brought to your attention in particular.

1) Both reviewers commented on the novelty of the approach to spinal cord injury treatment using IL-6 delivery and especially appreciated the section on adverse effects. However, they noted that the underlying reasons for the adverse effects are not well described.

2) Another concern about the adverse effects is the lack of a proper dose-response curve and the arbitrary choice of high and medium/low doses, rather than a pharmacologically informed approach.

3) The statistical analysis and details of experimental numbers, animal numbers, and repeats are important but missing throughout the study, such as in Figures 2 and 3.

4) Improvement of the images is required by providing high-magnification, high-resolution data with double/triple labeling for cellular localization.

5) Finally, the downstream molecular targets and cellular localization of STAT3 are unclear regarding the beneficial and adverse effects.

Reviewer #1

Authors investigation to determine the therapeutic potential of PDGF-B modified heat shock protein nanoparticles to induce post-stroke pericyte activation is interesting and well needed as pericytes play a crucial role in stroke prognosis. Use of modified nanoparticles, MRI, and flow cytometry added strength to the study. However, lack of approach to determine the therapeutic potential of the treatment on the functional outcomes and the acute end point of the study (3 days of stroke) limits overall scope of the study. Below are the comments that add strength to the manuscript.

Comments:

1. In the methods sections, "CB-17 mice were selected for their limited collateral circulation, which minimizes variability in infarct volume" and ischemia induction for 2 hours need more clarity.

2. Nanoparticle formulation was administered immediately after reperfusion, and the endpoint of the study is 3 days after reperfusion. It is crucial to specify the stability of this formulation and half-life after administration and any potential acute toxicity.

3. Please add clear sequence details of the T2-MRI.

4. Behavioral assessment within 3 days of stroke only with the Cylinder test is the weakest portion of this manuscript which drastically lowering the rigor on authors claims on "therapeutic potential of PDGF-B HSPNs"

5. Fig. 1 show fluorescent labelled PDGF-B HSPNs reached brain which is expected as stroke disrupts the BBB. It is crucial step here to demonstrate the uptake of this formulation by the brain cells in the ipsilateral cortex or strategy to determine the direct effect of this formulation on the brain cells.

6. Infarct volume assessed by MAP-2 staining is not adding anything to strengthen as authors used T2-MRI to determine infarct volume. If it is feasible, it is a good idea to extend T1-MRI with contrast to determine the BBB, which further support pericyte activation mechanisms.

Overall, this study needs additional strategies to determine pharmacokinetics of this modified nano formulation to confirm its direct effects after stroke. Need comprehensive functional assessment after stroke to support the therapeutic potential of PDGF-B HSPNs that acts through the activation of pericytes

Reviewer #2

This manuscript evaluates intravenous platelet-derived growth factor-B-modified heat shock protein nanoparticles (PDGF-B HSPNPs) administered immediately after reperfusion in a mouse tMCAO model and reports reduced infarct volume, improved motor outcome, and increased pericyte- and endothelial-marker-positive cell populations by flow cytometry and immunohistochemistry. The topic is of interest, and targeting pericyte PDGFRβ signaling could be therapeutically relevant. However, several issues limit the impact and interpretability of the work, particularly regarding novelty relative to prior work, rigor of the ischemia/reperfusion model verification, and incomplete methodological detail for key assays.

Major concerns

1) Novelty is incremental relative to prior work (Takagishi et al. 2021 eNeuro 8: eNEURO.0098-21.2021). The authors previously reported that PDGF-B HSPNPs reduced infarct volume and improved motor deficits when administered intravenously 24 h after tMCAO in mice, with mechanistic correlates including Akt phosphorylation and changes in neurotrophin-3, apoptosis, and astrogliosis. The present study shifts dosing to immediately after reperfusion and adds flow cytometry readouts of CD13/CD140b/CD31-positive populations. As written, the manuscript does not clearly articulate what new biological insight is gained beyond a different dosing time and additional marker quantification.

2) Limited generalizability due to choice of CB-17 strain (penumbra relevance). CB-17 mice were selected because limited collateral circulation reduces variability in infarct volume. While this can improve reproducibility, it also raises concerns about translational relevance and about whether the reported effects extend to commonly used strains (e.g., C57BL/6) with different collateral status and penumbral evolution.

3) Reperfusion confirmation is not described/verified. The methods describe occlusion by mechanical manipulation of a suture and "reperfusion" by reversal/removal after 2 h , but do not state how reperfusion was confirmed (e.g., laser Doppler/laser speckle flowmetry, or exclusion thresholds). Given that the intervention is administered "immediately after reperfusion," verifying reperfusion is essential for rigor and interpretation.

4) Brain dissociation / digestion for flow cytometry lacks critical detail. Flow cytometry is a central mechanistic component, but the dissociation protocol is insufficiently detailed: tissue was "treated with collagenase/dispase at 37{degree sign}C for 30 min" and then "treated with DNase" without specifying enzyme type/source, final concentrations (e.g., mg/mL or U/mL), volumes, buffer composition, trituration steps, filtration, stopping conditions. These details affect viability, endothelial/pericyte recovery, and marker integrity. Because pericytes are rare and sensitive, include gating strategy details and justification that the isolation does not bias against/for the populations of interest.

5) Claims/speculation about angiogenesis are not supported by data. The Discussion suggests PDGF-B HSPNPs "may promote angiogenesis" by recruiting pericytes and endothelial cells, yet the study does not directly assess angiogenesis or vascular remodeling. Without vessel-level endpoints, increases in CD31-positive events at day 3 could reflect multiple processes (cell survival, changes in activation state) rather than increased angiogenesis.

6) Please clarify whether the investigators were blinded for all endpoints and describe randomization procedures succinctly in the Methods (some of this appears in ARRIVE checklist but should be clear in the main text).

7) Please address the statistical power for flow cytometry and immunohistochemistry endpoints (notably small n for several measures; flow cytometry and CD13/PDGFRβ IHC appear to be N=3/group).

8) Please include representative gating plots and fluorescence-minus-one (FMO) controls for the multicolor panel, given the interpretation of "activated pericytes" as CD13⁺CD140b⁺ events.
